# A plasma miRNA signature for lung cancer early detection

**DOI:** 10.18632/oncotarget.22950

**Published:** 2017-12-05

**Authors:** Qixin Leng, Yanli Lin, Fangran Jiang, Cheng-Ju Lee, Min Zhan, HongBin Fang, Yue Wang, Feng Jiang

**Affiliations:** ^1^ Department of Pathology, University of Maryland School of Medicine, Baltimore, MD 21201, USA; ^2^ Departments of Biological Sciences, University of Maryland, Baltimore County, Baltimore, MD 21250, USA; ^3^ Departments of Epidemiology & Public Health, University of Maryland School of Medicine, Baltimore, MD 21201, USA; ^4^ Department of Biostatistics, Bioinformatics and Biomathematics, Georgetown University Medical Center, Washington, DC 20057, USA; ^5^ Department of Mathematics & Statistics, University of Maryland, Baltimore County, Baltimore, MD 21250, USA

**Keywords:** diagnosis, lung cancer, plasma, microRNA, biomarkers

## Abstract

The early detection of lung cancer continues to be a major clinical challenge. Using whole-transcriptome next-generation sequencing to analyze lung tumor and the matched noncancerous tissues, we previously identified 54 lung cancer-associated microRNAs (miRNAs). The objective of this study was to investigate whether the miRNAs could be used as plasma biomarkers for lung cancer. We determined expressions of the lung tumor-miRNAs in plasma of a development cohort of 180 subjects by using reverse transcription PCR to develop biomarkers. The development cohort included 92 lung cancer patients and 88 cancer-free smokers. We validated the biomarkers in a validation cohort of 64 individuals comprising 34 lung cancer patients and 30 cancer-free smokers. Of the 54 miRNAs, 30 displayed a significant different expression level in plasma of the lung cancer patients *vs.* cancer-free controls (all P < 0.05). A plasma miRNA signature (miRs-126, 145, 210, and 205-5p) with the best prediction was developed, producing 91.5% sensitivity and 96.2% specificity for lung cancer detection. Diagnostic performance of the plasma miRNA signature had no association with stage and histological type of lung tumor, and patients’ age, sex, and ethnicity (all p > 0.05). The plasma miRNA signature was reproducibly confirmed in the validation cohort. The plasma miRNA signature may provide a blood-based assay for diagnosing lung cancer at the early stage, and thereby reduce the associated mortality and cost.

## INTRODUCTION

About 155,870 Americans will die from lung cancer in 2017, more than the other 3 leading cancers combined (breast, prostate, and colorectal cancers). Worldwide 1.37 million deaths are attributed to lung cancer annually. Over 85% lung cancers are non-small cell lung cancers (NSCLC). NSCLC mainly consists of adenocarcinoma (AC) and squamous cell carcinoma (SCC). Tobacco smoking is the major cause of NSCLC. The disease is usually diagnosed at advanced stages when the prognosis is poor, resulting in an overall 5-year survival rate of approximately 14%[1]. However, the 5-year survival rate in patients with stage I NSCLC that has been surgically resected can be as high as 83%. Therefore, finding NSCLC earlier may reduce the mortality [1]. The early detection of lung cancer in a large randomized trial using low-dose CT (LDCT) has revealed a 20% reduction in mortality as compared to chest X-rays [2]. However, there are some limitations in using LDCT for lung cancer early detection and screening, including over-diagnosis, excessive cost and the harm associated with radiation exposure [3, 4]. Furthermore, most countries outside the US can’t afford to institute widespread CT screening, and many medical centers in the US do not yet follow guidelines. In addition, European studies show less significant results, and thus LDCT scan is not recommended for lung screening [5]. Therefore, the development of non-invasive or circulating biomarkers that can accurately and cost-effectively diagnose early stage lung cancer is required [6].

MicroRNAs (miRNAs) are a class of small non-coding RNAs (∼22-nt) that can regulate gene expression [7]. Dysregulation of some miRNAs has been found in relation to oncogenesis and tumor metastasis [8–11]. Importantly, plasma miRNAs directly released from primary tumors or the circulating cancer cells might provide biomarkers for malignancies [11]. We have been one of the first to show that miRNAs are highly stable in peripheral plasma, due to their small size and relative resistance to nucleases [10–14]. Using a microarray platform, we further identified three plasm miRNAs (miRs-21, 210, and 486-5p), which used in combination had 75% sensitivity and 85% specificity for diagnosis of stage I NSCLC [15]. To date, numerous plasma miRNAs have been identified that show the potential for distinguish lung cancer patients from non-cancer subjects [11, 16–21]. However, none of them has been accepted in the clinical settings for lung cancer diagnosis, mainly due to the low sensitivity and specificity.

Since next-generation deep sequencing (NGS) could analyze clinical specimens for detecting novel genes with high-throughput purposes and a wide detectable expression range [22], we recently used the whole-genomic NGS to define a miRNA profile of primary lung tumor tissues [23]. We successfully identified 54 lung cancer–related miRNAs (Supplementary Table 1) [23], which not only included the previously published lung cancer–related miRNAs [21, 24–28], but miRNAs that had not been identified as associated with lung cancer. The lung tumor-associated miRNAs defined by GNS may provide a comprehensive list of biomarker candidates for developing high quality circulating biomarkers of lung cancer. The objective of this study was to investigate whether the miRNAs defined by GNS could be used as plasma biomarkers for lung cancer with high sensitivity and specificity.

## RESULTS

### Developing miRNA biomarkers for lung cancer detection

**Figure 1:**
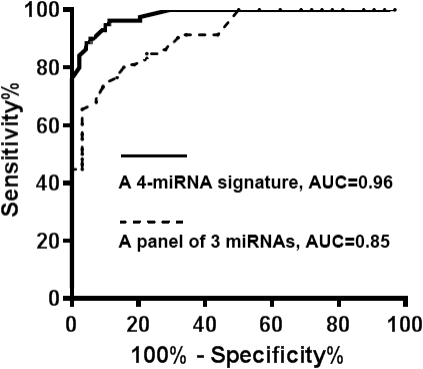
The comparison of the four-plasma miRNA signature and the panel of three miRNAs in a development cohort A prediction model based on four-plasma miRNA signature (miRs-126, 145, 210, and 205-5p) was developed for distinguishing lung cancer patients from cancer-free smokers. The ROC curve of the four-plasma miRNA signature produced an AUC of 0.96, yielding 91.5% sensitivity and 96.2% specificity. The ROC curve of panel of three miRNAs had an AUC of 0.85, producing 75.5% sensitivity and 85.3% specificity.

The 54 miRNAs had a <35 Ct value in plasma of all the subjects, implying that the miRNAs could be reliably measurable in peripheral plasma samples. Among the 54 miRNAs, 30 (55.6%) displayed a significantly different plasma expression level of lung cancer patients vs. cancer-free smokers (All p<0.05) (Table 1). The individual miRNAs exhibited AUC values of 0.52-0.80 in distinguishing lung cancer patients from cancer-free controls in the development cohort (Table 1). We used logistic regression models with constrained parameters as in LASSO based on ROC criterion to identify and optimize a panel of biomarkers. The four miRNAs (miRs-126, 145, 210, and 205-5p) were selected as the best biomarkers (all P <0.001) and incorporated into a logistic model: Probability of a lung cancer patient = e^x^/ (1 + e^x^), where x =. The logistic model produced 0.96 AUC for lung cancer detection (Figure 1). Furthermore, Pearson correlation among expression levels of the four plasma miRNAs was low (p> 0.05), implying that their diagnostic values were complementary to each other. Using Youden’s index, we set up optimal cutoff at 0.76. An individual tested with the signature, who had a cutoff ≥0.76, might be considered as a lung cancer patient. Subsequently, combined use of the four miRNAs as a signature by simply calculating the equation produced 91.5% sensitivity and 96.2% specificity. In addition, the plasma miRNA signature had significantly higher AUC (0.96 vs. 0.85) (Figure 1), sensitivity (91.5% vs. 76.0%), and specificity (96.2% vs. 85.3%) than did our previously developed three-plasma miRNA panel (all p<0.05) (Table 2). Moreover, including other miRNAs in the signature did not improve the accuracy for lung cancer diagnosis. The expression level of miR-205-5p was associated with SCC (p<0.05). The expression levels of miRs-210 and 205-5p were related with smoking pack-years (all p<0.05). However, using the four-plasma miRNA signature could diagnose lung cancer independent of histological type and stage of the NSCLC, and age, gender, and ethnicity of subjects (All p > 0.05), but their smoking pack-years (p < 0.05).

**Table 1:** The miRNAs having a different plasma expression level in lung cancer patients vs. cancer-free smokers of a development cohort

miRNAs	P-value	AUC (95% CI)	Sensitivity	Specificity
miR-422a	0.0319	0.5328 (0.4180 to 0.6476)	52.38	66.00
miR-326	0.0130	0.6236 (0.5312 to 0.7710)	61.11	62.96
miR-324-3p	0.0063	0.6825 (0.5772 to 0.7879)	63.49	66.67
miR-103a-3p	0.0109	0.6450 (0.5340 to 0.7559)	58.73	60.00
miR-30a-5p	0.0350	0.5815 (0.4752 to 0.7078)	57.14	60.00
miR-1285	0.0025	0.7380 (0.6252 to 0.8510)	66.67	66.67
miR-1254	0.0017	0.7068 (0.6516 to 0.8701)	71.73	70.00
miR-574-5p	0.0122	0.5921 (0.4786 to 0.7055)	50.79	53.33
miR-146b-5p	0.0210	0.6439 (0.5306 to 0.7572)	58.49	60.00
miR-27a-3p	0.0164	0.6344 (0.5227 to 0.7461)	57.14	73.33
miR-27b-3p	0.0286	0.5630 (0.4445 to 0.6815)	50.70	50.00
miR-222-3p	0.0152	0.6000 (0.4874 to 0.7126)	55.56	56.67
miR-106a-3p	0.0331	0.6587 (0.5497 to 0.7677)	60.32	66.67
miR-92a-3p	0.0012	0.7899 (0.7003 to 0.8796)	71.43	86.87
miR-29c	0.0040	0.6804 (0.5740 to 0.7868)	65.08	60.00
miR-24a-3p	0.0151	0.6619 (0.5538 to 0.7701)	60.32	60.00
miR-486-5p	0.0048	0.7957 (0.7066 to 0.8848)	70.97	83.33
miR-425-5p	0.0043	0.6529 (0.5445 to 0.7614)	60.32	70.00
miR-221-3p	0.0095	0.5758 (0.4616 to 0.6900)	57.14	51.72
miR-301a-3p	0.0138	0.6465 (0.5254 to 0.7654)	61.11	62.96
miR-148a	0.0253	0.6085 (0.4941 to 0.7228)	58.73	66.67
miR-148b	0.0197	0.5812 (0.4661 to 0.6963)	54.84	62.27
miR-193a-3p	0.0076	0.7111 (0.6016 to 0.8206)	68.25	66.67
miR-21	0.0370	0.5783 (0.4629 to 0.6937)	58.73	63.33
miR-19b-3p	0.0397	0.6619 (0.5520 to 0.7719)	68.25	63.33
miR-210-3p	0.0061	0.7254(0.6249 to 0.8259)	66.43	63.33
miR-145	0.0012	0.7233 (0.6198 to 0.82670)	71.43	71.33
miR-126-3p	0.0023	0.7767 (0.6736 to 0.8798)	71.43	73.33
miR-223-3p	0.0098	0.6000 (0.4874 to 0.7126)	53.97	56.67
miR-205-5p	0.0012	0.7268 (0.6099 to 0.8302)	71.43	71.33

Abbreviations: AUC, the area under receiver operating characteristic curve; CI, confidence interval.

**Table 2:** The comparison of the four-plasma miRNA signature and the panel of three miRNAs in a development cohort and a validation cohort

	A development cohort		A validation cohort	
	Sensitivity (95% CI)^*^	Specificity (95% CI)^*^	Sensitivity (95% CI)^*^	Specificity (95% CI)^*^
The four-plasma miRNA signature	91.56 (83.58 to 96.17)	96.23 (90.36% to 99.29%)	91.18 (76.32 to 98.14)	96.67 (82.78 to 99.92)
The panel of three miRNAs	75.46 (64.89 to 83.45)	85.26 (76.06% to 91.89%)	76.47 (58.83 to 89.25)	83.33 (65.28 to 94.36)

Abbreviations: CI, confidence interval.

^*^ P<0.05.

### Validating the plasma miRNA signature in a different cohort of cases and controls

The four miRNAs were successfully assessed in the validation cohort, and displayed a different plasma expression level between lung cancer patients vs. cancer-free individuals (All p<0.05). We used the optimal cutoff established in the above development cohort to determine diagnostic performance of the four-plasma miRNA signature. The plasma miRNA signature produced similar sensitivity (91.2%) and specificity (96.7%) in the validation cohort as did in the development cohort. Furthermore, the plasma miRNA signature generated higher sensitivity (91.2% vs. 76.5%) and specificity (96.7% vs. 83.3%) compared with the panel of the three miRNA biomarkers (All p<0.05) (Table 2). In addition, combined use of the four miRNAs had no statistical differences of sensitivity and specificity between stages and histological types of lung tumors (P>0.05). Moreover, the biomarker signature had no association with age, gender, and ethnicity of participants (All p > 0.05), except their smoking pack-years (p < 0.05). Altogether, the plasma miRNA signature was reproducibly validated in a different set of cases and controls for the early detection of lung cancer.

## DISCUSSION

So far, numerous circulating miRNA profiles in plasma and serum have been identified as noninvasive biomarkers for lung cancer [11, 16–21, 29–32]. However, there is a small overlap between the identified miRNA biomarkers, because of some reasons below: 1), the biomarkers were developed from the limited number of miRNA biomarker candidates. 2), the lack of correlation of miRNA expression levels between serum and plasma may lead to the differences of the biomarkers between the two liquid components of blood [33]. 3), the variation in pre-analytical factors, such as sample preparation procedures and different normalization strategies makes comparison between studies difficult.

To address the challenges, we systematically and comprehensively characterized a miRNA profile of NSCLC in surgically resected lung tumor tissues by using whole-transcriptome NGS [23]. We identified 54 miRNAs with a fold change (FC) ≥2.0 in NSCLC tissues vs. noncancerous tissues [23]. Interestingly, the identified miRNAs of lung cancer not only comprised these previously published lung cancer–related miRNAs including the ones discovered by The Cancer Genome Atlas [11, 16–21, 24–32], but also miRNAs whose changes had not been found in lung tumors. The miRNAs may provide potential high-quality biomarkers for lung cancer diagnosis. Furthermore, given that RNA released during the coagulation process may alter the composition of circulating miRNAs in serum, plasma may be the preferred sample choice [33]. Therefore, the primary emphasis of this study was to evaluate the comprehensive set of 54 miRNA biomarker candidates in plasma to develop more accurate and robust circulating biomarkers for lung cancer early detection.

To reduce the variation in pre-analytical factors in term of bias linked to sampling methods, storage and purification, in this study we collected blood and prepared plasma using the well-established SOPs developed by the NCI-EDRN [34, 35]. We used lysis solution to maximally reduce the possible contamination from RBCs in plasma ^13, 44-49^. To further diminish the bias linked to plasma quality, we tested the samples for hemolysis by measuring the free hemoglobin content using spectrophotometry [36]. Samples with absorbance peaks at 414 nm were considered positive for free hemoglobin and excluded from analysis. The plasma samples that were positive to these blood cells-related miRNAs were also excluded from the study. To further produce reliable results, we avoided repeated cycles of thawing and re-freezing plasma and RNA samples to diminish the degradation. Only RNA samples that had a 260/280 ratio of 1.8–2.0 and a RNA integrity number of ≥7 were analyzed. In addition, we used a well-established and validated qPCR assay [37] to quantify expression of the plasma miRNAs. As a result, our newly developed four-plasma miRNA signature created higher sensitivity and specificity for lung cancer detection than did the three plasma miRNA panel [38]. Furthermore, the diagnostic performance of the biomarkers was further blindly validated in a different cohort, suggesting that the plasma miRNA signature might be a robust assay for lung cancer diagnosis. In addition, the four miRNAs (miRs-126, 145, 210, and 205-5p) of the biomarker signature were not expressed in any type of blood cells [39]. Moreover, the performance of this plasma miRNA signature for lung cancer diagnosis was independent of tumor stage and histology. This new discovery in plasma might be an important characteristic if it is employed for more precisely and easily identifying early stage lung cancer.

Nadal et al. identified a four-miRNA signature (miRs-193b, 301, 141 and 200b) in serum for lung cancer detection with a sensitivity of 96% and a specificity of 95%[16]. In our present study all the four miRNAs also showed a different expression level in plasma of lung cancer patients vs. cancer-free smokers. However, our logistic regression models with constrained parameters as in LASSO analysis did not include the four serum miRNAs in our plasma miRNA signature. The differences of miRNAs between our plasma and Nadal’s serum signatures could partially be due to the lack of correlation of miRNA expression levels in the two different body fluids. Since RNA released during the coagulation process may alter the composition of circulating miRNAs in serum rather than plasma samples, plasma may be the preferred sample choice for the development of circulating biomarkers [33]. Furthermore, our analysis of the plasma signature by simply calculating the equation with the established cut-off would be a convenient tool in the clinics. Nonetheless, a new study for directly comparing the miRNA signatures of lung cancer patients in plasma and serum samples is needed, Dysregulation of the four miRNAs has been proven to associate with lung tumorigenesis. For example, change of circulating miR-126 could act as a significant biomarker in the prognosis of various cancers, including NSCLC [40]. miR-145 is dysregulated in lung cancer cells [41]. miR-145 inhibits lung cancer cell migration and invasion by targeting PDK1 via the mTOR signaling pathway [42]. miR-210 can regulate the hypoxic response of tumor cells [43–45]. We have reported that miR-210 overexpression in plasma is associated with lung cancer [46–48]. Elevated miR-205-5p expression participates in the development and progression of lung SCC [23, 49]. We have shown that miR-205-5p is one of three miRNAs that could be used as sputum biomarkers for the early detection of lung cancer [50, 51].

There are some limitations in this present study. 1), the plasma samples were obtained from the hospital-based patients with clinical diagnosis. The participants might not be representative of high-risk populations (e.g., heavy smokers) in screening setting for lung cancer. We will perform a prospective and multisite lung cancer screening trial to validate the diagnostic value of the plasma miRNA signature. 2), the NLST indicated that the early diagnosis of lung cancer by using LDCT could considerably reduce the mortality [4]. However, LDCT has a low specificity for the early detection of lung cancer, presenting a major clinical challenge [52]. We are evaluating whether the plasma miRNA signature could improve the specificity of LDCT for the early detection of NSCLC by specifically distinguishing malignant from benign pulmonary growths. 3), exploration of biomarkers in blood exosomal miRNA profiles has recently become a hot research topic [53]. Yet critical issues, including methods to specifically and cost-effectively isolate exosomal miRNAs, need to be well addressed before the exosomal miRNAs could be employed as noninvasive biomarkers [54]. We will perform a different study to compare the cell-free plasma and exosomal miRNA biomarkers to determine which would be better or if their combined use has synergistic efficiency for lung cancer detection. 4), based on patient and pulmonary nodule characteristics on CT images, several clinical predictive models have been developed to discriminate lung cancer from benign growths [55–58]. However, they only have moderate diagnostic performance. Furthermore, the assessments of cell-free circulating tumor DNA (ctDNA) or DNA methylation status of gene promoters have attracted increasing attention as potential liquid biopsy tests for lung cancer [59–61]. Our ongoing efforts are to compare the diagnostic efficiency of the plasma miRNA signature with those of the cell-free DNA biomarkers and clinical prediction models in the early detection of lung cancer.

In summary, a four-plasma miRNA signature that could accurately differentiate early stage NSCLC patients from cancer-free smokers was successfully developed and validated. Nevertheless, undertaking a prospective study to further validate this plasma miRNA signature in a prospective cohort is required.

## MATERIALS AND METHODS

### Patients and clinical specimens

This study and the related protocols were approved by the Institutional Review Boards (IRB) of University of Maryland Baltimore and Veterans Affairs Maryland Health Care System. Using the inclusion and/or exclusion criteria recommended by U.S. Preventive Services Task Force for lung cancer screening in heavy smokers [62], we recruited lung cancer patients and cancer-free smokers. Briefly, we enrolled heavy smokers between the ages of 55-80 who had at least a 30 pack-year smoking history and were former smokers (quit within 15 years). Exclusion criteria included pregnancy, current pulmonary infection, surgery within 6 months, radiotherapy within 1 year, and life expectancy of < 1 year. We collected blood in BD Vacutainer spray-coated K2EDTA Tubes (BD, Franklin Lakes, NJ) and prepared plasma using the standard operating protocols (SOPs) developed by The NCI-Early Detection Research Network (EDRN)[34, 35]. The blood samples from cancer patients were collected at the time of initial consultation, prior to definitive surgical management and/or adjuvant therapy. The specimens were processed within 2 hours of collection by centrifugation at 1,300 X g at for 10 minutes 4°C. We used red blood cell (RBC) lysis buffer to maximally reduce the possible contamination from RBCs in plasma [13, 44–49], which was immediately transferred to a fresh tube and stored at −80°C until use. A total of 126 NSCLC patients and 118 cancer-free smokers were recruited. Among the cancer patients, 44 were African American and 82 were Caucasian and 39 patients were female and 87 were male. Forty had stage I NSCLC, 43 with stage II, and 43 with stage III-IV. Of the cancer-free smokers, 41 were African American and 77 were Caucasian and 36 patients were female and 82 were male. There were no significant differences of age, race, gender and smoking status between the NSCLC patients and healthy individuals. In this study, the cases and controls were randomly grouped into two cohorts: a development cohort and a validation cohort. The development cohort consisted of 92 lung cancer patients and 88 cancer-free smokers, while the validation cohort comprised 34 lung cancer patients and 30 cancer-free smokers. The demographic and clinical variables of the two sets are shown in Tables 3–4.

**Table 3:** Characteristics of NSCLC patients and cancer-free smokers in a development cohort

	NSCLC cases (n = 92)	Controls (n = 88)	P-value
Age	66.39 (SD 10.26)	65.63 (SD 10.29)	0.32
Sex			0.36
Female	27	26	
Male	65	62	
Race			0.38
White	60	58	
African American	32	30	
Pack-years (median)	33.43	32.89	0.45
Stage			
Stage I	30		
Stage II	28		
Stage III-IV	34		
Histological type			
Adenocarcinoma	55		
Squamous cell carcinoma	37		

Abbreviations: NSCLC, non-small cell lung cancer.

**Table 4:** Characteristics of NSCLC patients and cancer-free smokers in a validation cohort

	NSCLC cases (n = 34)	Controls (n = 30)	P-value
Age	67.56 (SD 11.12)	64.48 (SD 11.25)	0.33
Sex			0.35
Female	10	9	
Male	24	21	
Race			0.39
White	22	20	
African American	11	10	
Pack-years (median)	34.28	31.36	0.32
Stage			
Stage I	10		
Stage II	9		
Stage III-IV	15		
Histological type			
Adenocarcinoma	21		
Squamous cell carcinoma	13		

Abbreviations: NSCLC, non-small cell lung cancer.

### RNA isolation

RNA was extracted from plasma by using a mirVana miRNA Isolation Kit (Ambion, Austin, TX) as described in our previous studies [12, 36, 38]. Purity and concentration of RNA were determined by using a dual beam UV spectrophotometer (Eppendorf AG, Hamburg, Germany). Integrity of RNA was determined by using a Bioanalyzer 2100 (Agilent Technologies, Santa Clara, CA).

### Quantitative reverse transcriptase PCR (qRT-PCR)

RT was carried out by using a RT Kit (Applied Biosystems, Foster City, CA) as described in our published works [12, 36, 38]. Briefly, RNA was applied for RT by using the Applied Biosystems 9700 Thermocycler (Applied Biosystems) according to the manufacturer’s instructions. The reaction comprised 50 nM stem–loop RT primer, 1x RT buffer, 0.25 mM each of dNTPs, and 3.33 U/μl MultiScribe reverse transcriptase in a total volume of 15 μL. Real-time PCR was performed to measure expressions of target miRNAs by using a PCR kit (Applied Biosystems) on a Bio-Red IQ5 Muilt-color Real-time PCR Detection System (Bio-Red, Hercules, CA). The 20 μl PCR reaction included RT product, 1x TaqMan^®^ Universal PCR Master Mix (Applied Biosystems), and the corresponding primers and Taqman probe for the target genes. The reactions were incubated in a 94-well plate at 95°C for 15 min, followed by 40 cycles of 95°C for 15 s and 60°C for 1 min. Expression levels of the 54 miRNAs in plasma were determined using comparative cycle threshold (Ct) method with the equation 2–ΔΔCt as previously described [12, 23, 28, 38, 50]. Ct values of the target miRNAs were normalized in relation to that of U6 [63]. For comparison, our previously identified panel of three plasma miRNA biomarkers (miRs-21, 210, and 486-5p) for lung cancer diagnosis [38] was also tested in the specimens by using the same protocol.

### Statistical analysis

To determine sample size, we used the area under receiver operating characteristic (ROC) curve (AUC) analysis and set the null hypothesis (H0) at 0.5. Accordingly, at least 28 subjects were required in each category of cases and controls to show a minimum difference of interest between an AUC of 0.75 versus an AUC of 0.5 with 80% power at the 5% significance level. Therefore, the development and validation cohorts of subjects in this present study would provide an enough statistical power for identification and verification of the biomarkers. Pearson’s correlation analysis was applied to assess relationship between plasma miRNA expressions and demographic and clinical characteristics of the patients and control individuals. The ROC curve and AUC analyses were used to determine sensitivity, specificity, and corresponding cut-off value of each miRNA [64]. To decide sensitivity and specificity, clinicopathologic results were used as the gold standard. We utilized logistic regression models with constrained parameters as in least absolute shrinkage and selection operator (LASSO) based on ROC criterion to eliminate the large number of irrelevant genes, develop composite panels of biomarkers, and optimize a diagnostic signature with the highest sensitivity and specificity. To compare the new signature and our previously developed three-plasma miRNA panel [38], we compared their AUCs to determine the sensitivity and specificity as previously described [56–58].

## SUPPLEMENTARY MATERIALS TABLE


