# Supplementary material for: A plasma miRNA signature for lung cancer early detection
Source: Oncotarget. 2017 Dec 5;8(67):111902–11. doi: 10.18632/oncotarget.22950 (PMC5762367; doi:10.18632/oncotarget.22950)
Supplement: Supplementary file 1 [file oncotarget-08-111902-s001.pdf]

A plasma miRNA signature for lung cancer early detection

SUPPLEMENTARY MATERIAL

Supplementary Table 1: Fifty-four miRNAs whose changes are associated with lung cancer defined by next-generation deep sequencing, and tested in plasma samples in this study*

mir-584-5p	mir-422a	mir-409-5p	mir-326	mir-324-3p	mir-122-5p	mir-103a-3p
mir-30a-5p	mir-1285	mir-1254	mir-574-5p	mir-146b-5p	mir-34b-3p	mir-27a-3p
mir-27b-3p	mir-10a-5p	mir-429	mir-222-3p	mir-125a-5p	mir-125b-5p	mir-124-3p
mir-106a-3p	mir-92a-3p	mir-29c	mir-24a-3p	mir-486-5p	mir-4753	mir-425-5p
mir-221-3p	mir-301a-3p	mir-200b-5p	mir-183-5p	mir-141	mir-204	mir-25
mir-195-5p	mir-152-3p	mir-148a	mir-148b	mir-19a-3p	mir-193a-3p	mir-205-5p
mir-21	mir-4251	mir-193b-3p	mir-19b-3p	mir-210-3p	mir-145	mir-126-3p
mir-155	mir-17-5p	mir-223-3p	mir-375	miR135a-5p		

*, the tested miRNAs of lung cancer not only comprise these previously published lung cancer–related miRNAs, including the ones discovered by The Cancer Genome Atlas (TCGA), but also miRNAs that have not been identified as associated with lung cancer.
